# Moderate vs. mild cases of overseas-imported COVID-19 in Beijing: a retrospective cohort study

**DOI:** 10.1038/s41598-021-85869-0

**Published:** 2021-03-22

**Authors:** Wenliang Zhai, Zujin Luo, Yue Zheng, Dawei Dong, Endong Wu, Zhengfang Wang, Junpeng Zhai, Yujuan Han, Huan Liu, Yanran Wang, Yaohui Feng, Jing Wang, Yingmin Ma

**Affiliations:** 1 Department of Emergency, Xuanwu Hospital, Capital Medical University, No.45 Changchunjie Street, Xicheng District, Beijing, 100053 China; 2 Department of Respiratory and Critical Care Medicine, Beijing Engineering Research Center of Respiratory and Critical Care Medicine, Beijing Institute of Respiratory Medicine, Beijing Chao-Yang Hospital, Capital Medical University, No. 5 Jingyuan Road, Shijingshan District, Beijing, 100043 China; 3 Department of Surgical Intensive Care Unit, Beijing Chao-Yang Hospital, Capital Medical University, Beijing, 100020 China; 4 Department of Radiology, Beijing Xiaotangshan Hospital, Beijing, 102211 China; 5 Department of Critical Medicine, Beijing Chest Hospital, Beijing, 101149 China; 6 Digestive Disease Center, Beijing Hospital of Traditional Chinese Medicine, Capital Medical University, Beijing, 100010 China; 7 Department of Endocrinology, Beijing Hospital of Traditional Chinese Medicine, Capital Medical University, Beijing, 100010 China; 8 General Internal Medicine, Beijing Xiaotangshan Hospital, Beijing, 102211 China; 9 Department of Health Education, Beijing Xiaotangshan Hospital, Beijing, 102211 China; 10 Department of Nephrology, Hospital of Capital Institute of Pediatrics, Beijing, 100020 China; 11 Department of Geriatrics, Beijing Hospital of Traditional Chinese Medicine, Capital Medical University, Beijing, 100010 China; 12 Beijing Youan Hospital, Capital Medical University, Beijing, 100069 China

**Keywords:** Infectious diseases, Viral infection, Public health

## Abstract

This study compared the differences in the clinical manifestations, treatment courses and clinical turnover between mild and moderate coronavirus disease 2019 (COVID-19). Clinical data of the patients with imported COVID-19 admitted to Beijing Xiaotangshan Designated Hospital between March 15 and April 30, 2020, were retrospectively analysed. A total of 53 COVID-19 patients were included, with 21 mild and 32 moderate cases. Compared with the mild group, the moderate group showed significant differences in breathing frequency, lymphocyte count, neutrophil percentage, neutrophil/lymphocyte ratio, procalcitonin, C-reactive protein, and dynamic erythrocyte sedimentation rate. In the moderate group, 87.5% exhibited ground-glass opacities, 14% exhibited consolidative opacities, 53.1% exhibited local lesions and 68.8% exhibited unilateral lesions. The proportion of patients who received antiviral or antibiotic treatment in the moderate group was higher than that in the mild group, and the number of cases that progressed to severe disease in the moderate group was also significantly higher (18.7% vs. 0%, p = 0.035). Compared with patients with mild COVID-19, those with moderate COVID-19 exhibited more noticeable inflammatory reactions, more severe pulmonary imaging manifestations and earlier expression of protective antibodies. The overall turnover of the moderate cases was poorer than that of the mild cases.

## Introduction

Population mobility between countries as well as between regions exacerbates the spread of COVID-19, resulting in unprecedented pressure from imported cases^1^. By 24:00 on June 2, 2020, a total of 1,762 overseas-imported cases had been reported in China, which were distributed in 28 provinces and cities in the country^2^. COVID-19 has become a global health emergency that seriously threatens human health.

Clinical manifestations of COVID-19 vary greatly, from the absence of symptoms to severe dyspnoea or even death. Old age, obesity, diabetes complications, hypertension, coronary artery disease and tumours are risk factors for COVID-19 aggravation and related death^3,4^. Populations with these characteristics have received great attention from scholars and clinicians. However, all populations are susceptible to SARS-CoV-2, and the number of non-severe patients is enormous^5^, which accounts for approximately 81% of the total number of COVID-19 patients. These non-severe patients are likely to be ignored due to milder symptoms, fewer complications, stronger body tolerance and fewer medical demands. Moreover, reports on the clinical characteristics, disease classification and disease turnover of these patients are relatively rare^6,7^.

Due to the complexity of COVID-19, experts have suggested classifying COVID-19 infection into mild, moderate, severe and critically severe types based on the clinical manifestations, CT imaging and laboratory outcomes of the patients^8,9^. As neither the mild type nor the moderate type belongs to an emergent condition, observational and treatment protocols for these patients are basically the same. Although CT imaging is able to differentiate between these two types, the selection of treatment protocols for these two types seems to have no direct relation with the classification outcomes. Whether it is necessary to differentiate patients with mild COVID-19 from those moderate disease according to CT images remains clinically complex.

Confronted with such complexity, as well as a lack of sufficient research on patients with non-severe COVID-19, we retrospectively analysed the clinical characteristics on admission, disease progression during treatment and clinical outcomes of patients with mild and moderate overseas-imported COVID-19, and the necessity of the differentiation between mild COVID-19 and moderate COVID-19 was also discussed.

## Results

### Clinical manifestations

**Figure 1:**
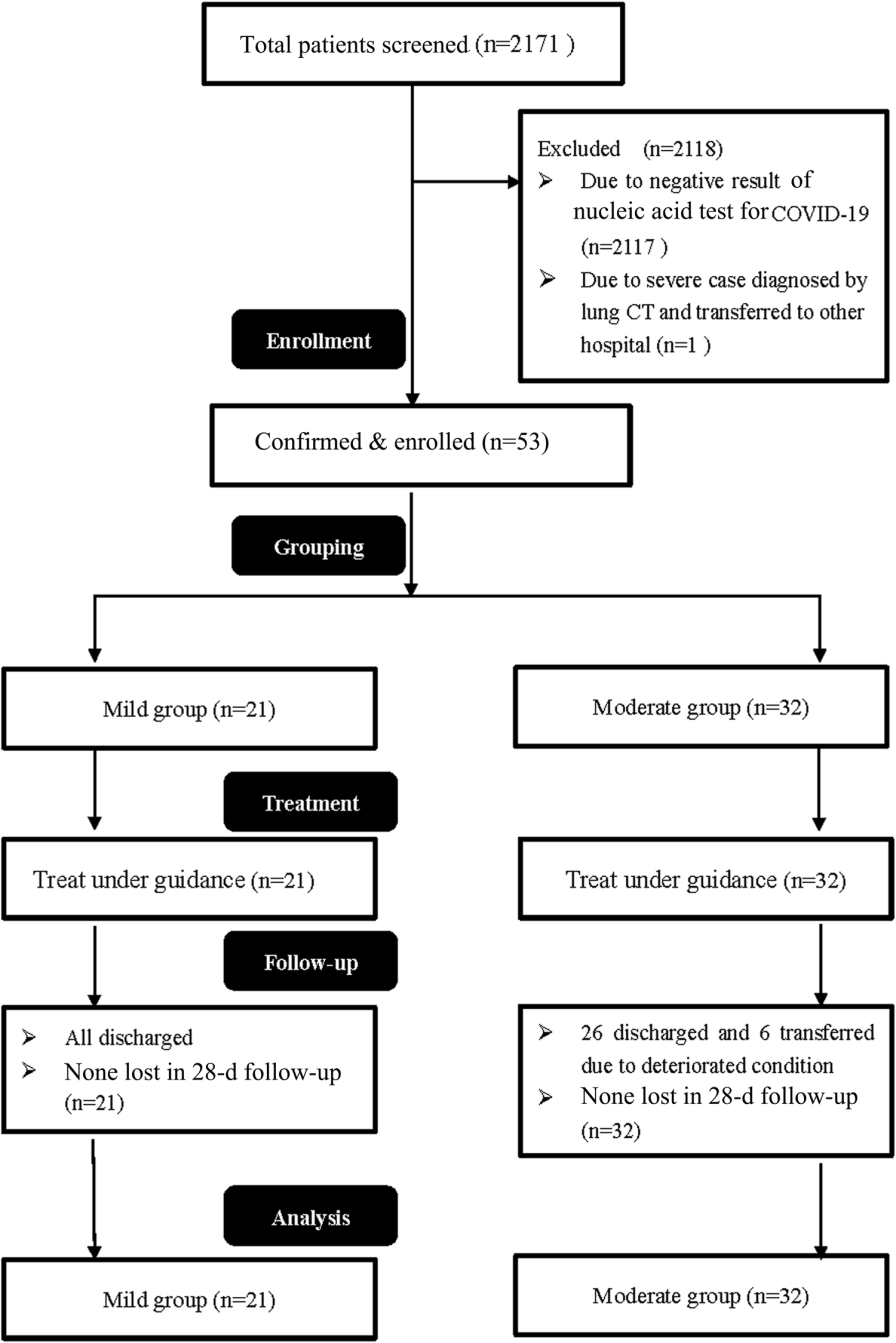
Cohort diagram of this study.

A total of 2171 suspected patients were screened, and 53 were finally confirmed with COVID-19 and hospitalized (2117 with a negative result and one with severe COVID-19 were excluded according to nucleic acid detection) (Fig. 1). The baseline data of the included patients are summarized in Table 1. All patients were imported from overseas, and 52.4% of the mild group and 46.9% of the moderate group were from the UK (P = 0.695), with the rest from France, America, Serbia, Spain and Thailand. The mild group and the moderate group had no significant differences in age, sex ratio, history of contact with confirmed or suspected COVID-19 patients, complications, or history of smoking.

**Table 1:** Clinical characteristics of the participants with COVID-19.

	Mild (n = 21)	Moderate (n = 32)	p
**Characteristic**			
Age, years	27[19.5–48]	23[20–33]	0.267^b^
**Sex, n(%)**			
Male	9(42.9)	12(37.5)	0.697^b^
Female	12(47.1)	20(62.5)	
**Epidemiology, n(%)**			
Returning from			
United Kingdom	11(52.4)	15(46.9)	0.695^b^
Others	10(47.6)	17(53.1)	
Exposure to confirmed or suspected patients	14(66.7)	18(56.2)	0.448^b^
**Comorbidities, n(%)**			
With chronic disease*	3(14.3)	4(2.5)	1.000^b^
Without chronic disease	18(85.7)	28(87.5)	
Current smoker, n(%)	6(28.6)	7(21.9)	0.579^b^
**Vital signs on admission**			
Temperature, °C	36.9[36.6–37.4]	37.05[36.8–37.3]	0.356^a^
Respiratory rate, TPM	18.0[18.0–19.0]	18.0[17.0–20.0]	**0.042**^a^
SpO_2_, %	97.0[96.0–98.0]	97.0[96.0–98.0]	0.767^a^
Systolic blood pressure, mmHg	123[105–128]	124[119–131]	0.107^a^
Heart rate, BPM	73.0[63.5–96.5]	69[65.25–77]	0.566^a^
Onset of symptoms to hospital admission, days	3.0[2.0–11.0]	4.5[2.0–8.0]	0.790^a^
**Severity status score**			
qSOFA ≥ 1	0[0–1]	0[0–1]	0.127^a^
SIRS	1[1–2]	1[0–2]	0.109^a^
APACHE II	2[0–2]	2[0–2]	0.977^a^
CURB-65 ≥ 1	0[0–0]	0[0–0]	0.345^a^
MEWS ≥ 3	2[1–3]	1.5[0–3]	0.093^a^
**Signs and symptoms on admission, n(%)**			
Fever	5(23.8)	15(46.8)	0.090^b^
Chills	2(9.52)	4(12.5)	1.000^b^
Cough	11(52.4)	17(53.1)	0.958^b^
Sputum	5(23.8)	6(18.8)	0.922^b^
Chest pain	2(9.52)	4(12.5)	1.000^b^
Dyspnoea	7(33.3)	6(18.8)	0.227^b^
Rhinobyon/rhinorrhoea	6(28.6)	12(37.5)	0.433^b^
Sore throat or dryness	12(57.1)	15(46.8)	0.465^b^
Headache/dizziness	8(38.1)	11(34.4)	0.782^b^
Malaise/fatigue	5(23.8)	3(9.38)	0.297^b^
Myalgia or arthralgia	4(19.0)	6(18.8)	1.000^b^
Nausea/vomiting/anorexia/diarrhoea	8(38.1)	8(25.0)	0.310^b^

*SpO*_*2*_ pulse oximeter O_2_ saturation; *qSOFA* quick sequential organ failure assessment; *SIRS* systemic inflammatory response syndrome; *APACHE II* acute physiology and chronic health evaluation II; *MEWS* modified early warning score; *TPM* times per minute; *BPM* beats per minute.

*Chronic diseases refer to chronic obstructive pulmonary disease, asthma, bronchiectasis, interstitial lung disease, hypertension, diabetes, and intrapulmonary or extrapulmonary malignancy.

^a^p according to the Wilcoxon rank sum test.

^b^p according to the χ^2^ test.

All bold values indicate significant differences at the level of 0.05 for a highlight purpose.

Fever, pharyngalgia or dry pharynx and tussiculation were the most common symptoms among the included patients. The moderate group showed a significantly higher breathing frequency in the resting state on admission than the mild group (18.0 [17.0–20.0] vs. 18.0 [18.0–19.0], P = 0.042).

### Laboratory and imaging examination

The count and percentage of lymphocytes in the moderate group were significantly lower than those in the mild group (P = 0.010 and P = 0.003, respectively; Table 2). The neutrophil percentage of the moderate group was significantly higher than that of the mild group (P < 0.001), whereas no significant difference in the neutrophil count was observed (P = 0.068). The NLR of the moderate group was significantly higher than that of the mild group (P < 0.001). Compared with the mild group, the moderate group also exhibited higher levels of ALT, creatinine, fibrinogen, PCT, CRP and ESR but a lower HDL level.

**Table 2:** Laboratory findings of patients with COVID-19.

Laboratory findings	Reference range	Mild (n = 21)	Moderate (n = 32)	p
**Blood cell count**				
White blood cell count, × 10^9^/L	3.5–9.5	5.1[4.05–5.2]	5.2[3.82–6.25]	0.894^a^
Neutrophil count, × 10^9^/L	1.8–6.3	2.11[1.69–3.23]	3.12[1.88–4.33]	0.068^a^
Neutrophil percentage, %	40–75	43.9[37.8–54.2]	57.3[48.25–64.7]	** < 0.001**^a^
Lymphocyte count, × 10^9^/L	1.1–3.2	2.25[1.62–2.85]	1.65[1.21–2.10]	**0.010**^a^
Lymphocyte percentage, %	20–50	44.0[35.8–50.05]	33.5[28.7–41.3]	**0.003**^a^
NLR		0.96[0.72–1.47]	1.74[1.19–2.18]	** < 0.001**^a^
Haemoglobin, g/L	130–175	134[127.5–141.5]	142.0[123.5–148.5]	0.467^a^
Platelet count, × 10^9^/L	125–350	210[191.5–247.0]	195.0[171.0–268]	0.743^a^
**Blood biochemistry**				
Albumin, g/L	40–50	44.4[43.25–46.55]	43.15[41.52–44.97]	0.072^a^
ALT, U/L	9–50	14.2[10.5–20.45]	18.1[6.51–12.75]	**0.029**^a^
AST, U/L	15–40	18.6[15.5–20.85]	20.5[17.2–24.85]	0.256^a^
Total bilirubin, mmol/L	1.7–23	10.13[6.84–12.28]	8.88[6.56–12.75]	0.611^a^
Potassium, mmol/L	3.5–5.3	4.20[4.12–4.72]	4.47[4.13–4.95]	0.317^a^
Sodium, mmol/L	137–147	139.6[138.25–141.55]	139.45[137.92–141.45]	0.849^a^
Creatinine, μmol/L	57–97	59.2[54.15–66.45]	67.7[58.67–91.27]	**0.033**^a^
LDH, U/L	120–250	163.30[143.25–199.15]	181.05[149.85–212.00]	0.252^a^
cTnI, pg/mL	0–40	0.1[0.1–0.1]	0.1[0.1–0.1]	0.418^a^
CKMB, U/L	0–25	8.8[8.0–12.1]	10.3[8.125–14.475]	0.287^a^
Triglyceride, mmol/L	0.6–1.7	0.68[0.54–1.01]	0.88[0.776–1.02]	0.052^a^
HDL, mmol/L	1.04–1.56	2.19[1.73–2.83]	1.45[1.13–2.42]	**0.008**^a^
LDL, mmol/L	1.03–3.38	2.21[1.65–2.42]	2.19[1.73–2.83]	0.335^a^
Total cholesterol, mmol/L	3.1–5.2	3.94[3.45–4.42]	3.80[3.25–4.61]	0.920^a^
Glucose, mmol/L	3.9–6.1	4.93[4.57–5.10]	4.92[4.72–5.19]	0.518^a^
Lactic acid, mmol/L	0–2	2.1[1.8–2.3]	2.1[1.7–2.4]	0.802^a^
**Coagulation**				
PT, s	9.1–12.1	13.6[13.35–14.15]	13.6[13.12–14.00]	0.964^a^
APTT, s	25.4–38.4	39.8[35.5–42.55]	39.15[37.00–45.22]	0.418^a^
Fibrinogen, mg/dL	2–4	2.62[2.495–3.015]	3.48[2.88–3.87]	** < 0.001**^a^
D-dimer, mg/L	0–231	220[220–300]	270[220–402.5]	0.127^a^
**Infection-related**				
PCT, ng/mL	0.02–0.5	0.03[0.03–0.375]	0.04[0.03–0.067]	**0.007**^a^
CRP, mg/L	0–6	0.39[0.165–1.335]	3.41[0.865–17.24]	**0.010**^a^
ESR, mm/h	0–15	7.0[4.0–10.5]	14.0[7.25–24.5]	**0.001**^a^
**Radiologic findings**				
GGO			28(87.5)	
Consolidation			14(43.8)	
Lesion distribution				
Local			17(53.1)	
Multifocal			15(46.9)	
Unilateral			22(68.8)	
Bilateral			10(31.3)	
Total CT score			2[1–3]	

Laboratory findings are presented as the median (25% quantile-75% quantile); radiologic findings are presented as the number of cases (percentage).

*NLR* neutrophil-to-lymphocyte ratio; *ALT* alanine aminotransferase; *AST* aspartate aminotransferase; *LDH* lactate dehydrogenase; *cTnI* cardiac troponin I; *CKMB MB* isoenzyme of creatine kinase; *HDL* high density lipoprotein; *LDL* low density lipoprotein; *PT* prothrombin time; *APTT* activated partial thromboplastin time; *PCT* procalcitonin; *CRP* C-reactive protein; *ESR* erythrocyte sedimentation rate; *GGO* ground glass opacity.

^a^p according to the Wilcoxon rank sum test.

All bold values indicate significant differences at the level of 0.05 for a highlight purpose.

**Figure 2:**
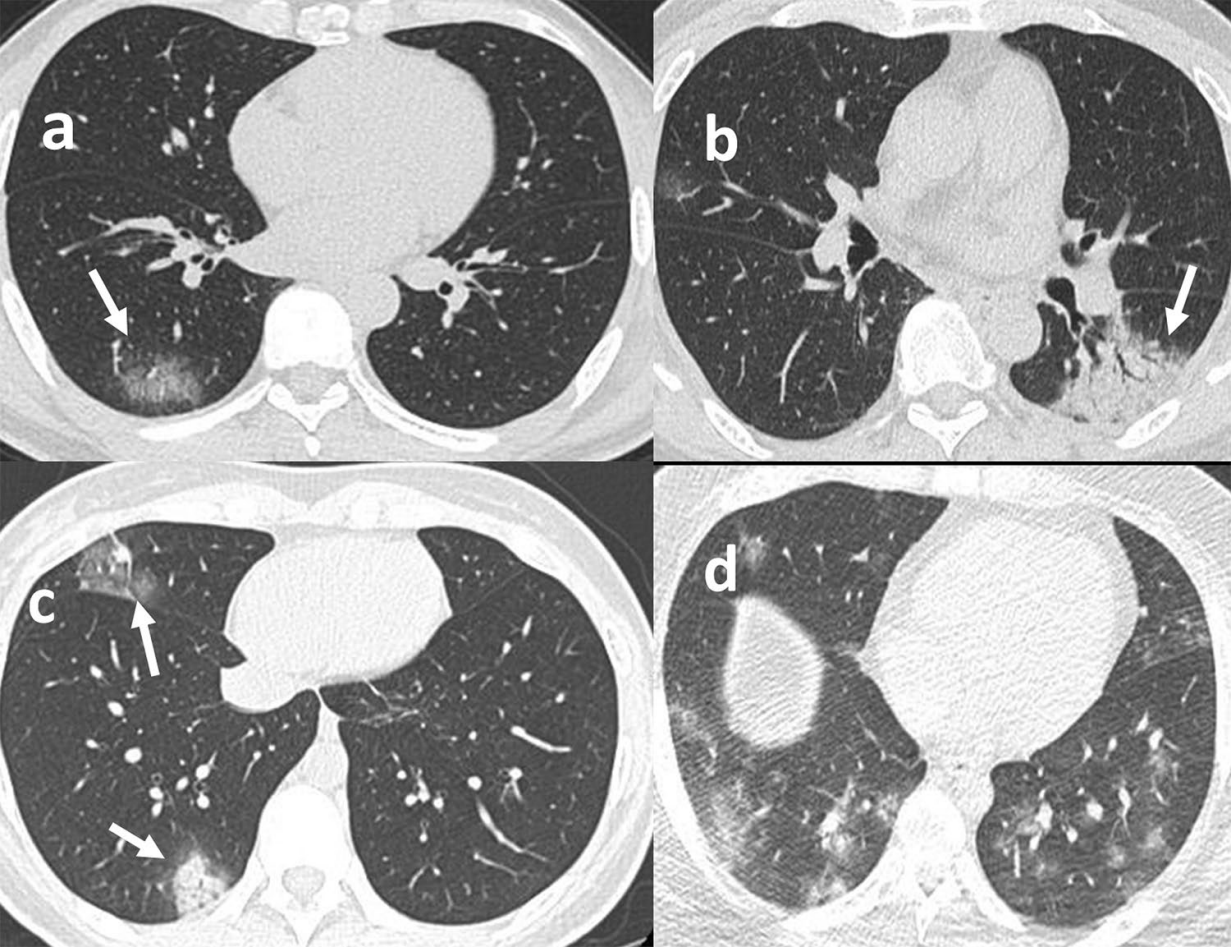
Chest CT findings of COVID-19 pneumonia. (**a**) GGO; (**b**) consolidation; (**c**) multifocal lesions; (**d**) bilateral lesions.

In the moderate group, 28 patients (87.5%) exhibited GGO, 14 (43.8%) exhibited consolidation, 17 (53.1%) exhibited local lesions and 22 (68.8%) exhibited unilateral lesions. Figure 2 shows some typical examples of the CT images. The median CT score of the moderate group was 2^1–3^. In contrast, the mild group did not exhibit characteristic changes according to the CT images.

### Treatment and outcomes

**Figure 3:**
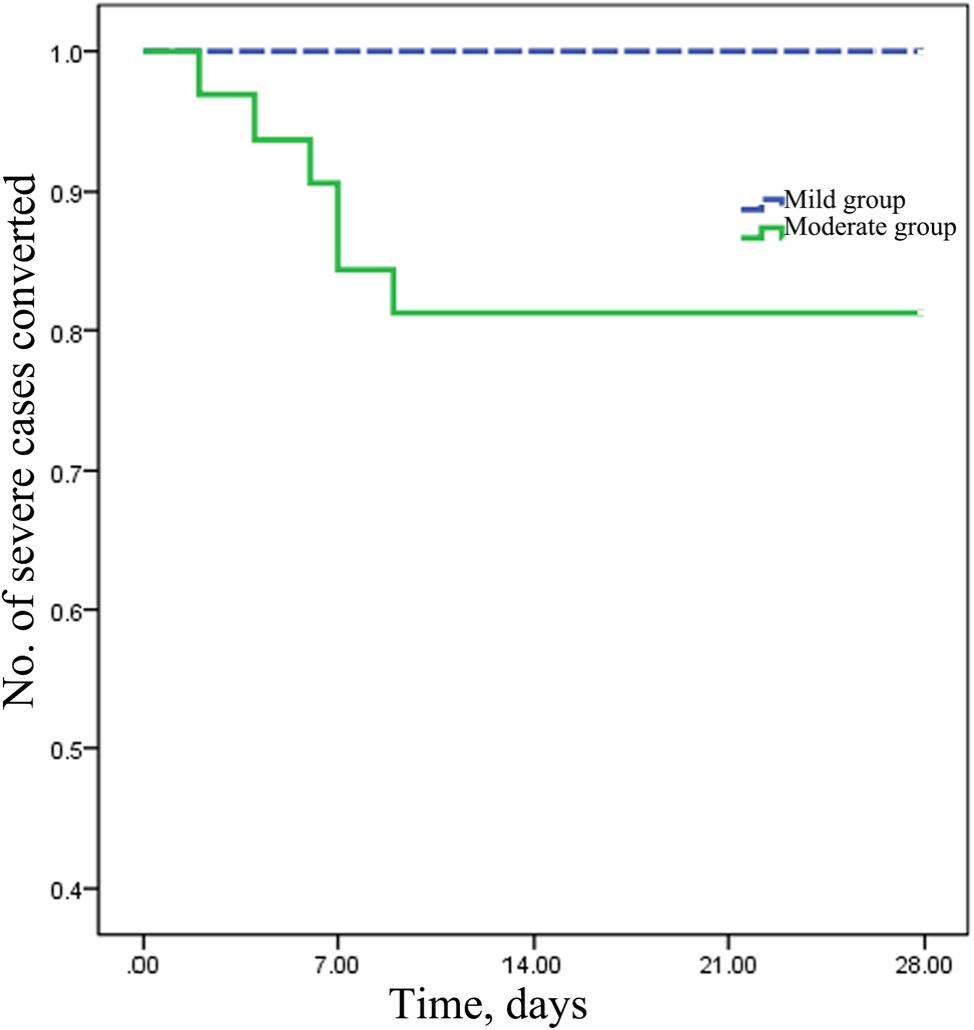
Kaplan–Meier survival analysis demonstrated increased severe case conversion in the moderate group (p < 0.043 by the log-rank test).

The proportion of patients who received chloroquine or arbidol antiviral treatment in the moderate group was higher than that in the mild group (Table 3). In the moderate group, 12 patients (37.5%) received oral administration of moxifloxacin or cefaclor for bacterial infection, whereas in the mild group, no patients received antibiotic treatment. Antibody detection at a 3-d interval showed earlier positive expression of IgG in the moderate group than in the mild group (P = 0.014). No characteristic emergence of positive IgM was detected (41 patients (77.3%) were not detected with positive IgM even by 28 d after discharge). During hospitalization, 6 patients in the moderate group exhibited aggravated pneumonia, which met the diagnostic criterion for severe pneumonia. These patients were subjected to intranasal oxygen inhalation and then transferred to other centres for further treatment. Among the 6 patients, 2 needed mechanical ventilation after the emergence of ARDS (Fig. 3), and 2 patients exhibited secondary infections, with one presenting with herpangina and the other complicated with influenza virus type A. Except for the 6 severe patients, all patients in the two groups were cured and discharged. The two groups did not show significant differences in the time span from disease onset to symptom improvement or the hospital stay. No sequelae were observed in the two groups at the time of discharge or at 28 d after discharge. Additionally, no significant difference in hospitalization costs was observed between the two groups (P > 0.05).

**Table 3:** Treatments and outcomes of patients with COVID-19.

	Mild (n = 21)	Moderate (n = 32)	p
**Treatments**			
Antiviral treatment	9(42.8)	30(93.7)	< 0.001^b^
Antibiotics	0	12(37.5)	0.004^b^
Nasal cannula oxygen therapy	0	6(18.7)	0.035^b^
**Complications**			
Acute respiratory distress syndrome	0	3(9.37)	
Secondary infection	0	2(6.25)	
**Outcomes**			
Severe COVID-19	0	6(18.7)	0.035^b^
Time to clinical improvement, days	10[3-22]	15[8–20]	0.720^a^
Time from illness onset to viral shedding, days	15[12–19]	12[9–17]	0.150^a^
Time from illness onset to positive serum immunoglobulin G, days	17.0[13.25–20.00]	13[10–16.5]	0.014^a^
Time from illness onset to positive serum immunoglobulin M, days	10[9–11]	11.5[2.25–14.75]	0.846^a^
Live discharge or transfer	21(100)	32(100)	
Transfer for advanced treatment	0	6(18.7)	0.035^b^
Hospital stay, days	14[11–21.5]	12.5[9.25–16]	0.106^a^
Hospital cost (× 10^3^ CNY)	12.1[8.5–18.3]	11.8[7.8–15.43]	0.604^a^
**Score on seven-category scale at discharge, n(%)**			
Not hospitalized, and resumed normal activities	21(100)	26(81.3)	
Not hospitalized but unable to resume normal activities	0	0	
Hospitalization, not requiring supplemental oxygen	0	2(6.25)	
Hospitalization, requiring supplemental oxygen	0	4(12.5)	
**Score on seven-category scale at day 28, n(%)**			
Not hospitalized, and resumed normal activities	21(100)	26(81.3)	
Not hospitalized but unable to resume normal activities	0	0	
Hospitalization, not requiring supplemental oxygen	0	2(6.25)	
Hospitalization, requiring supplemental oxygen	0	2(6.25)	
Hospitalization, requiring HFNC or noninvasive mechanical ventilation	0	0	
Hospitalization, requiring ECMO, invasive mechanical ventilation, or both	0	2(6.25)	
Death	0	0	

*ECMO* extracorporeal membrane oxygenation; *CNY* Chinese Yuan.

^a^p for the Wilcoxon rank sum test.

^b^p for the χ^2^ test.

### Intra-moderate group comparison

In this study, 6 patients with moderate COVID-19 exhibited disease aggravation during treatment, and they were compared with the remaining patients in the same group (n = 26). The results are summarized in Table 4.

**Table 4:** Laboratory findings of the moderate subgroups according to different prognoses.

Laboratory index	Reference range	Better prognosis (n = 26)	Worse prognosis (n = 6)	p
White blood cell count, × 10^9^/L	3.5–9.5	5.56[3.50–6.94]	4.20[4.10–5.53]	0.454^a^
Neutrophil count, × 10^9^/L	1.8–6.3	3.16[1.73–4.50]	2.74[1.95–3.94]	0.724^a^
Neutrophil percentage, %	40–75	56.3[47.45–63.10]	63.45[45.80–73.63]	0.351^a^
Lymphocyte count, × 10^9^/L	1.1–3.2	1.86[1.34–2.55]	1.13[0.82–1.72]	**0.033**^a^
Lymphocyte percentage, %	20–50	34.75[30.13–43.25]	25.35[15.93–42.03]	0.173^a^
NLR		1.62[1.15–2.06]	2.82[1.68–4.77]	**0.049**^a^
LDH, U/L	120–250	175.7[143.9–201.8]	206.6[181.7–300.4]	**0.041**^a^
CRP, mg/L	0–6	1.47[0.27–4.13]	20.92[15.04–52.20]	**0.002**^a^

Laboratory findings are presented as the median [25% quantile-75% quantile]; ^a^p according to the Wilcoxon rank sum test.

*NLR* neutrophil-to-lymphocyte ratio; *LDH* lactate dehydrogenase; *CRP* C-reactive protein.

These patients presented with a lower lymphocyte count than the patients whose prognoses were better (1.13 [0.82–1.72] vs. 1.86 [1.34–2.55]; p = 0.033). Among the 6 patients, 3 had lymphopenia, and 2 had lymphocyte counts near the lower threshold of the normal reference range at the time of admission. In addition, they presented with significantly higher NLR (2.82 [1.68–4.77] vs. 1.62 [1.15–2.06]; p = 0.049), CRP (20.92 [15.04–52.20] vs. 1.47 [0.27–4.13]; p = 0.002) and LDH (206.6 [181.7–300.4] vs. 175.7 [143.9–201.8]; p = 0.041).

## Discussion

COVID-19 patients exhibit varied symptoms. In this study, the initial symptoms of the non-severe imported cases could be fever and shivering, coughing and expectoration, chest distress and pain, dry and painful pharynx, myalgia and arthralgia, loss of appetite, diarrhoea, dizziness, headache, acratia and dispiritedness, anosmia, or ageusia, which were basically consistent with those reported in local cases^10,11^. Compared with the mild group, the moderate group did not show significant differences in the primary symptoms, except for a slightly higher proportion of the patients presenting with fever, which was similar to that reported by Sun^12^. The moderate group exhibited a faster breathing frequency in a resting state, which was the only different sign to differentiate moderate patients from mild patients. In terms of treatment and short-term prognosis, the demand for antiviral drugs and antibiotics in the moderate group was greater than that in the mild group in this study. In addition, all patients exhibiting exacerbated COVID-19 were from the moderate group. These findings suggest that early identification of patients with moderate COVID-19 should be needed and that different treatment methods should be adopted for them.

In this study, the variations in blood cells in the moderate group included a lower lymphocyte count, a decreased lymphocyte percentage, an increased NLR and an increased neutrophil percentage compared with those in the mild group. Zhang et al. reported a noticeable decrease in the count and percentage of lymphocytes in patients with severe COVID-19, and thus, they recommended using these changes as important warning signals for disease exacerbation^13^. The variations in lymphocytes observed in this study suggest that the influence of the virus on the immune function of the patient exists at an early stage rather than being limited to severe cases. To date, the underlying mechanisms and dynamics of the cellular immunologic response after SARS-CoV-2 infection remain unknown. SARS-CoV-2 infection causes cytokine storms and reduced lymphocytes (particularly CD4 + and CD8 + T cells), as well as suppression of CD4 + T cell production of IFN-γ, which might be associated with the severity of COVID-19^14^. An increase in the NLR was another noticeable variation in the moderate group in this study. The NLR is a well-known marker for systemic inflammation and infection and has been utilized as a predictive index for bacterial infection (including pneumonia). The change in the NLR is associated with the severity of COVID-19^15–17^. In this study, patients with moderate COVID-19 presented with an increased neutrophil count (although the difference was not significant; p = 0.068) and an elevated neutrophil percentage compared with those of patients with mild COVID-19. These phenomena might be consequences either of aggravated inflammation and stress response^18^ or of the mutual effect of innate immunity and adaptive immunity in vivo^19^. Changes in the classification of leukocytes may reflect the beginning of inflammation and immune reactions in vivo, which also affects patients’ clinical outcomes. Elevated pro-inflammatory factors, such as TNF-α and IL-6, are closely associated with reduced lymphocytes, and severe lymphocyte reduction tends to result in non-resolution of inflammation^20^, an increased ICU hospitalization rate^21^ and increased mortality. Lymphocyte reduction is particularly striking among patients who die during hospitalization^22,23^.

The inflammatory reaction level after SARS-CoV-2 infection also affects patients’ prognoses. In this study, the levels of CRP, PCT, fibrinogen and ESR in the moderate group were higher than those in the mild group. These findings were consistent with those reported in the literature^24^, which indicate that inflammatory reactions occur at different stages of COVID-19 progression and that inflammatory reactions in moderate COVID-19 are stronger than those in the mild type. Increased CRP and D-dimer and decreased fibrinogen were some of the primary characteristic abnormalities^25^. In this study, however, the fibrinogen level increased in the moderate group compared with that in the mild group. Presumably, in Zheng et al.’s study^25^, severe COVID-19 complicated with coagulation disorders led to disseminated intravascular coagulation in some patients, and a decreased fibrinogen level was a depletion manifestation. In this study, the level of D-dimer, as a fibrinolytic product, did not noticeably increase in the moderate group, and the moderate patients did not present with coagulation disorders of the coexistence of coagulation and hyperfibrinolysis, which suggested that fibrinogen did not enter a depletion state. Fibrinogen has been proven to be a biological marker of the severity of pneumonia, and its increase is one of the manifestations of SARS-CoV-2-induced inflammation^26,27^. In addition, in the moderate group, the PCT level and neutrophil percentage were higher than those in the mild group. An increased PCT level occurred in 3%-35% of COVID-19 patients^28,29^. Generally, an increase in the PCT level suggests bacterial infection rather than viral infection. Approximately 6%-15% of COVID-19 patients exhibited complications of infection of bacteria and fungi, and 20% were complicated with other respiratory tract infections^30–33^. Whether the increased PCT level in the moderate group of this study was related to complications caused by other pathogens remains to be verified.

The emergence of specific and neutralizing antibodies might be able to prevent the invasion of the virus into host cells; however, antibodies cannot be detected in all infected patients^34^. In this study, all patients with moderate COVID-19 were positive for IgG antibody before hospital discharge, whereas IgG positivity was not detected in 9 patients in the mild group, even by the end of the follow-up. Among all included patients, only 12 were positive for IgM antibodies. Similar findings have also been reported by Lee et al.^35^. In this study, the median time span from disease onset to the emergence of positive IgG in the moderate group was 13 d, which was earlier than that in the mild group (17 days). However, such a difference was not observed in IgM. Previous studies have shown that IgG positivity appears at 10–15 d after SARS-CoV-2 infection^32,34^. In addition, positive antibody expression appeared at 1–2 w after disease onset; severe and critically severe patients exhibited a higher IgM level and a lower IgG level than mild patients^36^. Patients with severe COVID-19 presented with a strong antibody response within 2 w after disease onset, and the antibody titre values in these patients were significantly higher than those in patients with non-severe COVID-19, which suggested that antibodies might be generated within 2 w after infection^37^. Although we found that the emergence of positive IgG in the moderate group occurred earlier than that in the mild group, we did not quantitatively analyse the antibody level. Overall, the production of antibodies is the consequence of the joint influence of the degree of inflammatory reactions and the autoimmune response, and a decreased antibody level in critically severe patients might be associated with a compromised immune response. To date, the significance of antibody emergence remains unclear. The emergence of antibodies has no direct relation with virus removal, and some COVID-19 patients may even exhibit long-term coexistence of positive antibodies and viral PCR results, which brings confusion and worry to the accuracy of clinical judgement and the value of vaccine research^38^.

In this study, 87.5% of the moderate cases exhibited GGO according to CT imaging, most of whom presented with a unilateral single focus. This finding was consistent with that reported in the literature^39,40^. However, Shi et al.^41^ and Bernheim et al.^42^ reported that most COVID-19 patients from Wuhan, China, exhibited multiple bilateral lesions. This inconsistency might be due to the younger age and non-severe disease of the patients involved in this study. In terms of dynamic imaging outcomes, COVID-19 patients present with a peak of variations approximately 10 d after disease onset, which suggests that the CT findings of COVID-19 are dynamic; therefore, timely rechecks are helpful for accurate and objective understanding of the progression of the disease^43^.

In addition, in the moderate group of this study, 6 patients presented with disease aggravation during treatment. The analysis of these patients would be helpful for clinicians to identify cases at potential risk of aggravation into severe and critically severe types in a timely manner. Our analytical results showed that most of these patients already had lymphopenia or a lymphocyte count near the lower threshold of the reference range at the time of admission; moreover, these cases were complicated with significantly higher NLRs and CRP and LDH levels than the remaining moderate cases. These findings indicated more severe inflammatory reactions and tissue injury in these patients. According to the literature^20,21^, the LDH levels of patients with severe and critically severe COVID-19 are significantly increased, whereas those of patients with non-severe COVID-19 remain at a normal level. Although our findings did not show a significant difference in LDH between the mild cases and the moderate cases, they did suggest that non-severe cases with increased LDH at an early stage had the risk of aggravation into severe and critical types.

This study has some limitations. First, it was conducted at a single centre and retrospective. With the alleviation of the epidemic situation in Beijing, the number of confirmed patients is significantly reduced. For this reason, the sample size of this study was relatively small. Second, the follow-up time of this study was short. Third, all patients involved in this study were imported cases, and most of them were young. These features of the patients might cause bias in the explanation of the obtained results. Last, the qualitative detection of antibodies in this study could not reflect the absolute antibody levels in vivo.

In conclusion, patients with moderate COVID-19 show more noticeable inflammatory reactions, more pulmonary imaging characteristics and earlier protective antibody expression than mild patients. Their turnovers are worse. Early identification of different types of COVID-19 followed by adoption of different treatment therapies is useful for predicting the progression of the disease and realizing reasonable allocation of medical resources.

## Patients and methods

### Study design

A total of 53 patients with imported COVID-19 who received treatment at Beijing Xiaotangshan Designated Hospital between March 16 and April 30, 2020, were consecutively included in this study.

The procedures of this study were performed in accordance with the Declaration of Helsinki and approved by the Ethics Committee of Beijing Xiaotangshan Designated Hospital (2020-LSD-04). Because COVID-19 infections were subject to emergent public health events, written informed consent was waived by the committee.

### Subjects

COVID-19 was diagnosed by detecting SARS-CoV-2 RNA in throat swab specimens with a virus nucleic acid detection kit (Shanghai BioGerm Medical Biotechnology Co., Ltd., Shanghai, China)^8^. Based on vital signs, symptoms, complications and CT imaging outcomes, the cases were classified into mild, moderate, severe and critically severe types. The mild type was defined as mild clinical symptoms without pneumonia manifestations according to imaging examination. The moderate type was defined as fever and respiratory symptoms with pneumonia manifestations. Severe COVID-19 was defined as a case that met any of the following criteria: (1) shortness of breath with a respiration rate (RR) ≥ 30 times/min; (2) oxygen saturation in a resting state ≤ 93%; (3) arterial partial pressure of oxygen (PaO_2_)/oxygen concentration (FiO_2_) ≤ 300 mmHg (1 mmHg = 0.133 kPa); and (4) progression of the lesions within 24–48 h > 50% according to pulmonary imaging examination. Critically severe COVID-19 was defined as a case meeting any of the following criteria: (1) respiratory failure, for which a ventilator is necessitated; (2) shock; and (3) complications of functional failure of other organs, for which intensive care unit (ICU) monitoring and treatment are needed.

### Treatment and data collection

The detailed data of the non-severe patients were collected, which included demographic information, medical history, history of COVID-19-related contact, symptoms, signs, severity assessments on admission and clinical manifestations. Peripheral blood and arterial samples on admission and during hospitalization were collected. The measurement indices included the neutrophil count, neutrophil percentage, lymphocyte count, lymphocyte percentage, neutrophil/lymphocyte ratio (NLR), haemoglobin concentration, blood platelet count (PLT), blood gas analysis, alanine transaminase (ALT), aspartate aminotransferase (AST), lactate dehydrogenase (LDH), total bilirubin, creatinine, high density lipoprotein (HDL), low density lipoprotein (LDL), total cholesterol, glucose, cardiac troponin I, lactic acid, prothrombin time (PT), activated partial thromboplastin time (APTT), fibrinogen, D-dimer, C-reactive protein (CRP), procalcitonin (PCT), and erythrocyte sedimentation rate (ESR). SARS-CoV-2-specific antibodies (serum immunoglobulin G (IgG) and immunoglobulin M (IgM)) were qualitatively detected. All patients were subjected to low-dose CT scanning on admission. The recorded data also included laboratory examination outcomes, imaging examination outcomes, and treatment methods, such as antivirals, antibiotics and breathing support, as well as the patient’s state at discharge. The survival state and self-care status of the patients were followed up via phone contact at 28 d after discharge. Their hospital stay and costs were also calculated.

### CT image review

All CT images were independently reviewed by two experienced radiologists. Imaging variations were defined as follows: (1) ground-glass opacification (GGO), hazy increased lung attenuation with preservation of bronchial and vascular margins; and (2) consolidation, opacification with obscuration of margins of vessels and airway walls. The degree of involvement of each lung lobe was classified based on the following criteria: (1) 0%; (2) minimal, 1%–25%; (3) mild, 26%–50%; (4) moderate, 51%–75%; and 5) severe, 76%–100%. According to the involvement, 0 (0%), 1 (minimal), 2 (mild), 3 (moderate), and 4 (severe) points were assigned to the lobe. Finally, the overall score of the lungs was obtained by summing the scores of the five lobes, which ranged from 0 to 20 points^39^.

### Statistical analysis

Data were processed with SPSS 22.0. Continuous variables are presented as the median and interquartile range (IQR), and their distribution normality was tested using the Kolmogorov–Smirnov method. Categorical data are presented as the number of cases and percentage. The rank sum test was used for continuous variables, and the chi-square test or Fisher exact test was used for categorical variables. P < 0.05 was considered statistically significant.

## References

[1] Zhang XA (2020). Importing coronavirus disease 2019 (COVID-19) into China after international air travel. Travel Med. Infect. Dis..

[2] The latest situation of the new coronavirus pneumonia epidemic as of 24:00 on June 2. http://www.nhc.gov.cn/xcs/yqtb/202006/2b49053eb2bd43b199865fe254a02fdc.shtml. Accessed June 3, 2020.

[3] Zhang G (2020). Clinical features and short-term outcomes of 221 patients with COVID-19 in Wuhan, China. J. Clin. Virol..

[4] Kalligeros M (2020). Association of obesity with disease severity among patients with COVID-19. Obesity (Silver Spring).

[5] Wu Z, McGoogan JM (2020). Characteristics of and important lessons from the coronavirus disease 2019 (COVID-19) outbreak in China: summary of a report of 72 314 cases from the Chinese Center for Disease Control and Prevention. JAMA.

[6] Feng Y (2020). COVID-19 with different severity: A multi-center study of clinical features. Am. J. Respir. Crit. Care Med..

[7] Wu J (2020). Clinical characteristics of imported cases of COVID-19 in Jiangsu Province: A multicenter descriptive study. Clin. Infect. Dis..

[8] National Health Commission of the People’s Republic of China. *Diagnosis and Treatment of Pneumonia Caused by SARS-COV-2 (Version 7)*. http://www.nhc.gov.cn/xcs/zhengcwj/202003/46c9294a7dfe4cef80dc7f5912eb1989/files/ce3e6945832a438eaae415350a8ce964.pdf. Accessed May 8, 2020.

[9] Clinical management of severe acute respiratory infection (SARI) when COVID-19 disease is suspected Interim guidance 13 March 2020. https://www.who.int/publications-detail/clinical-management-of-severe-acute-respiratory-infection-when-novel-coronavirus-(ncov)-infection-is-suspected. Accessed May 8, 2020.

[10] Li LQ (2020). COVID-19 patients' clinical characteristics, discharge rate, and fatality rate of meta-analysis. J. Med. Virol..

[11] Lechien JR (2020). Clinical and epidemiological characteristics of 1,420 European patients with mild-to-moderate coronavirus disease 2019. J. Intern. Med..

[12] Sun L (2020). Clinical features of patients with coronavirus disease 2019 (COVID-19) from a designated hospital in Beijing, China. J. Med. Virol..

[13] Zhang G, Zhang J, Wang B, Zhu X, Wang Q, Qiu S (2020). Analysis of clinical characteristics and laboratory findings of 95 cases of 2019 novel coronavirus pneumonia in Wuhan, China: A retrospective analysis. Respir. Res..

[14] Chen G (2020). Clinical and immunological features of severe and moderate coronavirus disease 2019. J. Clin. Invest..

[15] Berhane M (2019). The role of neutrophil to lymphocyte count ratio in the differential diagnosis of pulmonary tuberculosis and bacterial community-acquired pneumonia: A cross-sectional study at Ayder and Mekelle Hospitals, Ethiopia. Clin. Lab..

[16] Qin C (2020). Dysregulation of immune response in patients with COVID-19 in Wuhan, China. Clin. Infect. Dis..

[17] Yang AP (2020). The diagnostic and predictive role of NLR, d-NLR and PLR in COVID-19 patients. Int. Immunopharmacol..

[18] Kong M, Zhang H, Cao X, Mao X, Lu Z (2020). Higher level of neutrophil-to-lymphocyte is associated with severe COVID-19. Epidemiol. Infect..

[19] Liu X (2016). Prognostic significance of neutrophil-to-lymphocyte ratio in patients with sepsis: a prospective observational study. Mediat. Inflamm..

[20] Heffernan DS (2012). Failure to normalize lymphopenia following trauma is associated with increased mortality, independent of the leukocytosis pattern. Crit. Care.

[21] Fan BE (2020). Hematologic parameters in patients with COVID-19 infection. Am. J. Hematol..

[22] Wang D (2020). Clinical characteristics of 138 hospitalized patients with 2019 novel coronavirus-infected pneumonia in Wuhan, China. JAMA.

[23] Terpos E (2020). Hematological findings and complications of COVID-19. Am. J. Hematol..

[24] Wang L (2020). C-reactive protein levels in the early stage of COVID-19. Med. Mal. Infect..

[25] Zheng C (2020). Risk-adapted treatment strategy for COVID-19 patients. Int. J. Infect. Dis..

[26] Hopstaken RM, Cals JW, Dinant GJ (2009). Accuracy of lipopolysaccharide-binding protein (LBP) and fibrinogen compared to C-reactive protein (CRP) in differentiating pneumonia from acute bronchitis in primary care. Prim. Care. Respir. J..

[27] Han H (2020). Prominent changes in blood coagulation of patients with SARS-CoV-2 infection. Clin. Chem. Lab. Med..

[28] Liu F (2020). Prognostic value of interleukin-6, C-reactive protein, and procalcitonin in patients with COVID-19. J. Clin. Virol..

[29] Zhang JJ (2020). Clinical characteristics of 140 patients infected with SARS-CoV-2 in Wuhan, China. Allergy.

[30] Goyal P (2020). Clinical characteristics of covid-19 in New York City. N. Engl. J. Med..

[31] Zhou F (2020). Clinical course and risk factors for mortality of adult inpatients with COVID-19 in Wuhan, China: A retrospective cohort study. Lancet.

[32] Rawson TM (2020). Bacterial and fungal co-infection in individuals with coronavirus: A rapid review to support COVID-19 antimicrobial prescribing. Clin. Infect. Dis..

[33] Kim D (2020). Rates of co-infection between SARS-CoV-2 and other respiratory pathogens. JAMA.

[34] Nie Y (2020). (2020). Neutralizing antibodies in patients with severe acute respiratory syndrome-associated coronavirus infection. J. Infect. Dis..

[35] Lee YL (2020). Dynamics of anti-SARS-Cov-2 IgM and IgG antibodies among COVID-19 patients. J. Infect..

[36] Hou H (2020). Detection of IgM and IgG antibodies in patients with coronavirus disease 2019. Clin. Transl. Immunol..

[37] Zhao J (2020). Antibody responses to SARS-CoV-2 in patients of novel coronavirus disease 2019. Clin. Infect. Dis..

[38] Wang B (2020). Long-term coexistence of SARS-CoV-2 with antibody response in COVID-19 patients. J. Med. Virol..

[39] Chung M (2020). CT imaging features of 2019 novel coronavirus (2019-nCoV). Radiology.

[40] Wang M (2020). Typical radiological progression and clinical features of patients with coronavirus disease 2019. Aging (Albany NY).

[41] Shi H (2020). Radiological findings from 81 patients with COVID-19 pneumonia in Wuhan, China: A descriptive study. Lancet Infect. Dis..

[42] Bernheim A (2020). Chest CT findings in coronavirus disease-19 (COVID-19): relationship to duration of infection. Radiology.

[43] Pan F (2020). Time course of lung changes at chest CT during recovery from coronavirus disease 2019 (COVID-19). Radiology.

